# Performance and combustion characteristics of an HCCI engine fueled with n‑Butanol/diethyl ether blends under varying intake‑air temperatures

**DOI:** 10.1038/s41598-026-44203-2

**Published:** 2026-03-14

**Authors:** Radhwan Ali, H. Serdar Yücesu, Alper Calam, Hamit Solmaz

**Affiliations:** 1 Graduate School of Natural and Applied Sciences, Automotive Engineering Department, Gazi University, 06500 Ankara, Turkey; 2 Mechanical Engineering Department, Faculty of Engineering, University of Misan, Al Amarah, Iraq; 3 Automotive Engineering Department, Faculty of Technology, Gazi University, Ankara, Türkiye Turkey; 4 Gazi University, Technical Sciences Vocational High School, Ankara, Türkiye Turkey

**Keywords:** Butanol/DEE fuel blends, HCCI combustion, Intake air temperature (IAT), Engine performance, Emission characteristics, Energy science and technology, Engineering, Environmental sciences

## Abstract

This study was conducted to elucidate the combined effects of intake-air temperature (IAT), excess air ratio (λ), and fuel blend composition on the combustion behavior of an HCCI engine. Three butanol/diethyl ether blends (B15, B30, and B45) were systematically evaluated at a constant engine speed of 1000 rpm and a compression ratio of 12. The IAT was varied between 35 °C and 65 °C in 15 °C increments, while different λ values were applied to each blend to capture a broader spectrum of operating conditions. The findings demonstrate clear differences in combustion behavior and performance among the blends. Specifically, increasing the diethyl ether content in the B15 blend, together with higher IAT, advanced in-cylinder pressure development, heat-release rate, start of combustion, and CA50. It also provided the widest stable λ operating range, although PRRₘₐₓ reached 14 bar/°CA at rich conditions, exceeding the knock safety limit. In contrast, relative to the B15 blend, a butanol fraction of 45% retarded ignition timing while achieving optimal combustion phasing between 7° and 11° after TDC. This shortened the combustion duration by approximately 59%, improved indicated thermal efficiency by nearly 20%, and reduced knocking by about 70%. The blend also achieved the highest IMEP of 6.27 bar, maintaining cyclic variability below 10% and ensuring stable combustion even at elevated IAT values. Additionally, the lowest emission levels were observed for the B15 blend at 65 °C, with CO and HC concentrations of 0.065% and 171 ppm, respectively, whereas CO₂ emissions showed the opposite trend, increasing as CO decreased. Overall, the results identify B45 as the most effective blend for maximizing efficiency and combustion stability, while B15 provides the broadest λ operating window, highlighting a measurable trade-off between efficiency optimization and operating flexibility in HCCI engines.

## Introduction

The ongoing depletion of fossil fuel reserves, coupled with the severe environmental impacts of their combustion, has intensified the global pursuit of sustainable and efficient energy alternatives. Among all energy-consuming sectors, transportation accounts for a significant share of fossil fuel use and remains a dominant source of greenhouse gas emissions and urban air pollution^1–3^. In response, researchers and policymakers have increasingly focused on electrification technologies, including hybrid-electric and fully electric vehicles, as potential pathways toward cleaner mobility. However, despite their promise, limited battery capacity, long charging times, and the lack of a well-developed charging infrastructure still constrain their large-scale deployment^4,5^. Consequently, extended-range hybrid systems integrating electric propulsion with an internal combustion engine as an auxiliary power unit have emerged as a practical transition technology toward sustainable transportation. Among advanced combustion technologies, the homogeneous charge compression ignition (HCCI) concept offers high thermal efficiency and ultra-low emissions, making it a promising candidate for hybrid applications^6^. Nevertheless, the major limitation of HCCI engines is their narrow and unstable operating range, primarily due to difficulties in controlling combustion phasing^7^. One practical approach to address this limitation is to modify fuel properties, particularly by blending fuels with complementary physical and chemical characteristics.

N-butanol is an alcohol-based fuel, classified as a second-generation biofuel, that can be produced from lignocellulosic feedstocks or via the ABE fermentation pathway^8^. This production pathway not only uses non-food biomass but also minimizes competition with food crops, unlike first-generation biofuels derived from edible sources, which can affect food prices^9^. In addition to these environmental benefits, n-butanol has a high octane rating, making it particularly suitable for HCCI engines. These properties enhance combustion phasing control, promote stable operation without knocking at high engine loads, and support efficient regulation of the heat release rate in HCCI combustion^10^. Compared to other alcohol fuels, n-butanol has a higher energy content and greater blending stability, showing greater miscibility with gasoline and diesel than branched or lower-carbon alcohols^11^. Siva et al.^12^ carried out a comprehensive numerical and modeling study to investigate the combustion and emission characteristics of a direct‑injection HCCI‑CI engine fueled with diesel/butanol blends. The findings of their study showed that adjusting four principal parameters, including the compression ratio, EGR, injection pressure, and timing, can significantly lower soot and NOx emissions and reduce the indicated specific fuel consumption across various fuel blend ratios. Uyumaz^13^ investigated the influence of blending butanol and isopropanol in various proportions with n-heptane in an HCCI-SI engine. The results indicated that, for n-heptane to function effectively as a low-reactivity fuel in HCCI operation with a CR of 13, it should be mixed with higher-reactivity components and the intake temperature raised. Moreover, increasing the butanol fraction in the blend delayed the HCCI combustion phase, thereby providing a practical approach to controlling combustion phasing. Solmaz et al.^14^ conducted an HCCI combustion analysis using the CONVERGE-CFD simulation tool to examine the effects of blending oxygenated alcohols with n-heptane. The results revealed that blends containing 10% alcohol experienced knocking under high-load conditions, while increasing the alcohol proportion, especially with butanol, improved efficiency and combustion stability by reducing the maximum pressure rise rate, leading to smoother combustion. Gainey et al.^15^ experimentally examined the autoignition characteristics of various alcohol fuels under HCCI operating conditions. The findings showed that n-butanol exhibited the highest reactivity, distinctive combustion behavior, and reduced sensitivity to intake temperature at low boost pressures, indicating partial cool flame reactivity. Conversely, the other alcohols displayed similar autoignition trends with minor variations in reactivity, suggesting their potential interchangeability with minimal impact on ignition behavior. Kireç et al.^16^ conducted an experimental investigation on n-butanol/n-heptane blends in an HCCI engine, comparing their performance and combustion characteristics with those of neat n-heptane across various compression ratios and λ. The incorporation of n-butanol moderated oxidation kinetics and shifted the heat-release process closer to top dead center, thereby improving control of combustion phasing and partially suppressing knock, which is a significant limitation in HCCI combustion. Consequently, an ITE of 35–44% was achieved, along with reductions in CO and HC emissions. Although neat n-heptane exhibited the broadest operating range, increasing the CR effectively extended the operating range of the n-butanol blends.

Diethyl ether (DEE) is an oxygenated additive recognized for its superior capability to enhance the ignition quality of base fuels compared to alcohol-based additives. Its high cetane number, excellent fuel solubility, low self-ignition temperature, and adequate energy density have been highlighted by numerous researchers in recent decades^17–20^. It exists as a liquid under normal ambient conditions, which enhances its suitability for storage and handling as a fuel. It is commonly synthesized by the dehydration of ethanol^21^. In addition to these favorable properties, DEE has been used not only as a blending component with liquid fuels but also with gaseous fuels such as hydrogen, LPG, and biogas, as well as with diesel and various biodiesel blends^22^. Banke et al.^23^ experimentally examined fuel-rich methane combustion in a single-cylinder HCCI engine with a CR of 10. The findings revealed that DEE was the most effective additive, enabling stable operation at nearly a 20% mass fraction. DEE facilitated earlier heat release and exhibited a less pronounced negative temperature coefficient than n-heptane and DME, thereby enhancing ignition propensity and improving overall combustion stability. Polat et al.^24^ investigated the influence of fusel oil/DEE blends on the combustion behavior and emission characteristics of an HCCI engine. Their results revealed that increasing the DEE fraction enhanced in‑cylinder pressure and HRR, thereby extending the stable operating range. The DEE40 blend improved the IMEP by 67.5% while reducing HC and CO emissions, whereas the DEE60 blend achieved the highest ITE of 42.5%. However, further increasing the DEE proportion to 80% led to higher CO and HC emissions. Ardebili et al.^25^ optimized the operating parameters and emission characteristics of an HCCI engine fueled with a fusel oil/DEE blend using the RSM. Their findings revealed that a DEE proportion of about 42% yielded optimal performance by enhancing combustion stability, advancing CA50, and improving torque, MPRR, and BSFC. At the same time, emissions of NOx, UHC, CO₂, and CO were significantly reduced, confirming the effectiveness of DEE as an additive for improving HCCI combustion characteristics. Sudheesh et al.^26^ investigated the use of DEE as an ignition improver for biogas‑fueled HCCI engines. The results demonstrated that HCCI operation with a biogas/DEE fuel combination enabled a wider load range and consistently delivered higher brake thermal efficiency (BTE) than both spark-ignition (biogas) and dual-fuel (biogas/diesel) modes. Furthermore, HC emissions under HCCI operation were significantly lower than those observed in the SI mode, highlighting the advantages of the biogas/DEE HCCI strategy.

HCCI combustion is constrained by its narrow operating range, in which misfires occur at low loads and knock at high loads, due to the lack of precise control over autoignition and combustion phasing. Fuel properties alone, such as octane and cetane numbers, are insufficient to regulate spontaneous oxidation reactions and, therefore, cannot fully satisfy the operational requirements of HCCI engines. To overcome these limitations, numerous studies have investigated control strategies designed to extend the limited operating range of HCCI combustion^27–29^. Among these, adjusting IAT has proven particularly effective for optimizing combustion phasing and enhancing overall performance, as demonstrated in several previous investigations^30^. Hence, the chemical reactions in HCCI mode depend on factors such as the initial reactant temperature, which plays a critical role in determining ignition timing, heat-release characteristics, and the formation of both intermediate and final combustion products^31,32^. Building on the authors’ previous work^33^, this study addresses a clear gap in the HCCI literature. Although butanol and DEE have been investigated separately in such engines, the interaction between butanol/DEE blends and IAT variation has not been systematically explored. Therefore, this work experimentally evaluates butanol/DEE fuel blends at different intake air temperatures while maintaining a constant engine speed and compression ratio and varying the excess air ratio (λ) to establish comprehensive operating maps. The novelty of this study lies in providing the first systematic assessment of the coupled effects of fuel blending and IAT on combustion phasing, engine performance, and emission characteristics. The analyzed parameters include in-cylinder pressure, IMEP, PRRmax, HRR, CA10, CA50, combustion duration (CD), indicated thermal efficiency (ITE), and CO and HC emissions.

## Materials and methods

### Test fuels and blending procedure

In this study, various butanol/DEE blend fuels were prepared as test fuels. The butanol ratios in the DEE mixtures were selected as 15%, 30%, and 45% by volume (B15, B30, and B45, respectively) to represent low, medium, and high butanol contents. The blends were homogenized using an IsoLAB ultrasonic homogenizer for 1 h to ensure thorough mixing and complete miscibility of the components. After homogenization, the blends were stored in sealed glass containers at ambient temperature (approximately 20–25 °C) for 72 h. During this period, no visible phase separation, sedimentation, or stratification was observed, confirming the stability of the mixtures. This procedure ensured consistent and reliable fuel properties throughout the experiments. Their volumetric blending ratios are presented in Table 1, and the chemical properties, obtained from a commercial supplier, are listed in Table 2. The prepared fuel blends were then employed in HCCI engine trials to systematically evaluate their effects on combustion phasing, thermodynamic efficiency, and exhaust emission profiles under controlled operating conditions.

**Table 1:** Composition and designation of butanol/DEE fuel blends.

Abbreviation	Volumetric Percentage of Fuel Components
B15	15% n-butanol 85% Diethyl ether
B30	30% n-butanol 70% Diethyl ether
B45	45% n-butanol 55% Diethyl ether

**Table 2:** Properties of butanol and DEE Fuels^34–37^.

Formula	Butanol	DEE	B15	B30	B45
	C_4_H_9_OH	C_4_H_10_O	-	-	-
Boiling point (°C)	117	34.6	47	59.3	71.7
Octane number (-)	96–98	-	-	-	-
Auto-ignition temperature (°C)	355	160	189	219	248
Density (Kg/m³)	810	713.4	728	742	757
Cetane number (-)	17–25	< 125	110	95	80
Lower heating value (MJ/Kg)	36	33.9	34.2	34.5	34.8
Percentage of oxygen by mass (%)	21.6	21.6	21.6	21.6	21.6
Heat of vaporization (KJ/kg)	430	360	371	381	392
Stoichiometric Air–Fuel Ratio	11.17	11.28	11.26	11.25	11.23
Viscosity (cSt)	2.63	0.23	0.59	0.95	1.31
Molecular weight[kg/kmol]	74	74	74	74	74

### Engine configuration and experimental setup

**Fig. 1:**
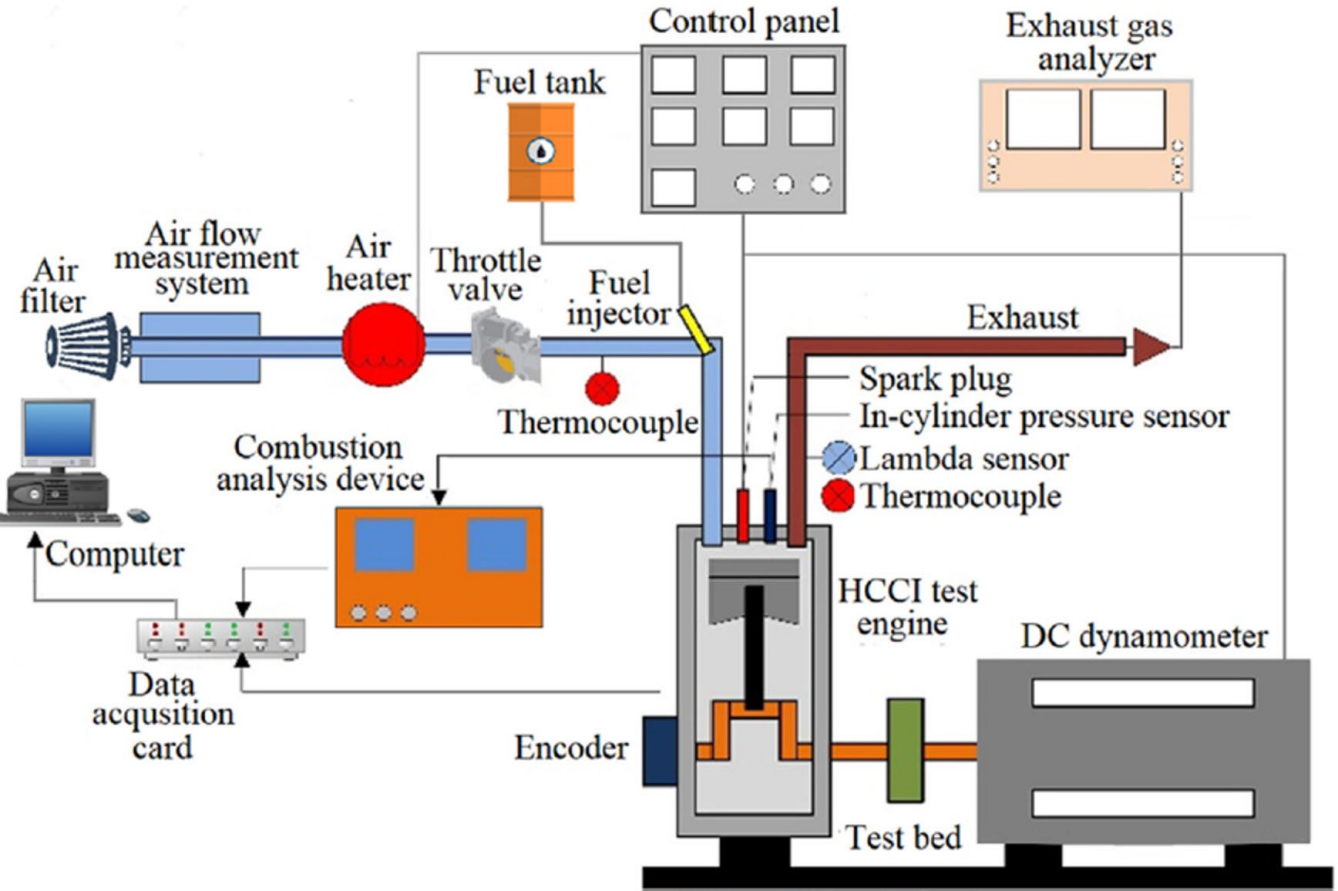
Schematic diagram of the HCCI engine experimental setup.

The experiments were conducted on a four-stroke HCCI-SI engine with a 450 cc displacement. The engine was water-cooled, naturally aspirated, and equipped with a port-injection system. A McClure DC-type dynamometer, rated at 30 kW at 6500 rpm, was integrated into the test setup to impose controlled mechanical loading and to facilitate high-resolution acquisition of engine torque and speed. Key engine parameters, including intake air temperature, engine speed, injection pulse, and coolant and oil temperatures, were monitored and controlled via the dynamometer control panel. Fuel delivery was regulated using a potentiometer integrated into the control panel, which adjusted the injector opening time and thereby controlled the air–fuel ratio. Initially, the engine was operated in spark-ignition mode for at least 15 min until the coolant and lubricating oil temperatures stabilized at 85 °C and 90 °C, respectively, to ensure steady operating conditions. All subsequent tests were conducted under these stabilized temperature conditions. Once these temperatures were reached, the spark-ignition system was deactivated, and the engine was switched to HCCI mode for the experimental investigations. However, the test engine could not operate in HCCI mode with the standard camshaft configuration. To mitigate this limitation, the intake and exhaust valve lifts were modified to 3.5 mm and 5.5 mm, respectively. These modifications enhanced in-cylinder thermal stratification and promoted residual gas retention, thereby enabling the fuel–air mixture to reach its auto-ignition threshold within the compression stroke. The engine test setup, including the measurement systems, is depicted in Fig. 1, and the engine’s specifications are summarized in Table 3. Additionally, the estimated uncertainties of the experimental measurements, expressed as percentages, along with the measurement accuracy of each instrument, are presented in Table 4. In-cylinder pressure was recorded using a Kistler 6121 piezoelectric sensor with a measurement range of 0–250 bar, a sensitivity of 14.7 pC/bar, and an accuracy of ≤ 0.5%. Pressure measurements were averaged over 50 consecutive cycles to minimize cycle-to-cycle variation for each test condition. The raw signals were first amplified using a Cussons P4110 combustion analyzer and then digitized through a National Instruments USB-6259 data acquisition system. To accurately correlate the pressure data with piston position, an Opkon encoder with a 0.36° crank-angle resolution was installed on the crankshaft to monitor and maintain the TDC, crank angle (CA), and engine speed. The encoder signal was synchronized with the in-cylinder pressure data through the data acquisition card and recorded on the computer. Additionally, the engine was fitted with a manifold heater to regulate the intake conditions, and the IAT was continuously monitored using a K-type thermocouple. During the experiments, the IAT was systematically varied from 35 °C to 65 °C in increments of 15 °C. In parallel, exhaust gas concentrations were continuously measured using a Bosch BEA350 analyzer, providing high-precision readings of O₂, NOₓ, CO, and HC. The analyzer’s specifications are summarized in Table 5, and it was calibrated by Bosch Türkiye prior to the experiments. The engine was operated at a fixed CR of 12:1 and a constant speed of 1000 rpm, while λ was systematically varied to facilitate a comparative assessment of the tested fuel blends across a broad operating range. Under these conditions, stable HCCI operation with minimal knocking and misfire was achieved, ensuring reliable autoignition.

**Table 3:** Specifications and technical details of the experimental engine.

Brand of engine	Ricardo hydra
Engine type	water‑cooled, naturally aspirated
Compression ratio	12:1
Bore × Stroke (mm)	80.26 × 8.90
Cylinder number	Single cylinder
Max. speed (rpm)	5400
Fuel supply method	(PFI) Port fuel injection
Max. power output (kW)	15
Valve configuration	1 intake and 1 exhaust

**Table 4:** The uncertainty and the accuracy of the tests and devices.

Lambda	Uncertainties [%]	Accuracy
	± 1.85	± 0.001
Torque (Nm)	± 0.24	± 0.2 (%)
Test fuels (g)	± 0.22	± 0.001 (g)
Engine speed (rpm)	± 1	-
IMEP (bar)	± 2.32	-
Start of combustion (CA)	± 1.25	-
Combustion duration (CA)	± 1.25	-

**Table 5:** Specifications of the exhaust gas measuring device.

Carbon monoxide (%)	Sensitivity	Range
	± 0.001	0 − 10
Nitrogen oxide (ppm)	± 1	0 − 5000
Hydrocarbon (ppm)	± 1	0 − 9999
Lambda (-)	± 0.001	0.5 − 9.999
Oxygen (%)	± 0.01	0 − 22

### Methodology of combustion analysis

Approximately 300 experiments were conducted, and the resulting datasets were used to generate comparative figures illustrating the effects of fuel blend composition and IAT on HCCI combustion characteristics. However, direct utilization of raw experimental data is often impractical; mathematical formulations were employed to process and interpret the results. In this study, a custom MATLAB algorithm was developed to analyze the combustion process. The algorithm was specifically developed using in-cylinder pressure data obtained from the data acquisition system to calculate key combustion parameters, including HRR, CA10, CA50, ITE, MPRR, and CD. The HRR was computed using the first law of thermodynamics, assuming a closed thermodynamic system with negligible mass exchange or gas leakage from the combustion chamber. The analysis accounted for transient heat-transfer interactions between the in-cylinder gas and the surrounding cylinder walls, thereby ensuring accurate resolution of the evolution of combustion energy throughout the cycle. Subsequently, the crank-angle-resolved HRR was computed using Eq. 1.

1 $$\frac{dQ}{d\theta}=\frac{k}{k-1}P\frac{dV}{d\theta}+\frac{k}{k-1}V\frac{dP}{d\theta}+\frac{d{Q}_{heat}}{d\theta}$$

Where $dQ$ represents the net heat release, while $V$ represents the cylinder volume and $P$ the in-cylinder pressure. $d\theta$ corresponds to the crank angle, and (*k*) is the specific heat ratio.$\frac{dQ_{heat}}{d_{\theta}}$ Represents the heat transferred from the gas to the cylinder walls.

The ITE was calculated using Eq. 2.

2 $${{\upeta}}_{th}=\frac{{W}_{net}}{{\dot{m}}_{butanol}{\times{Q}}_{LHVbutanol}+{\dot{m}}_{DEE}{\times{Q}}_{LHVDEE}}$$

Where $W_{net}$ represents the net work, $\dot{m}$denotes the cyclic fuel consumption, and ${\mathrm{Q}}_{\mathrm{L}\mathrm{H}\mathrm{V}}$is the lower heating value of the respective fuels (butanol and DEE).

The net work output of the HCCI engine was determined using Eq. 3.

3 $$W=\oint{Pdv}$$

Where $dV$ represents the change in cylinder volume, and $P$ corresponds to the change in in-cylinder pressure.

Equations 4 and 5 were used to determine $IMEP$ and ${PRR}_{max}$.

4 $$IMEP=\frac{{W}_{net}}{{V}_{strok}}$$

Where the ${W}_{net}$, refers to the net work and ${V}_{strok}$, the cylinder swept volume.

5 $${PRR}_{max}={\left[\frac{dp}{dCA}\right]}_{max}$$

Where, $\frac{dp}{dCA}$ Represents the maximum rate of change of the in-cylinder pressure with respect to the crankshaft angle.

The cyclic deviations were quantified using Eqs. 6,

6 $${COV}_{imep}=\frac{{\sigma}_{imep}}{\stackrel{-}{X}}\times100$$

Where, ${\sigma}_{imep}$: the standard deviation of the IMEP calculated over 50 consecutive engine cycles. $\stackrel{-}{X:}$ Represents the corresponding mean value of the effective pressure.

## Results and discussion

### In-cylinder pressure and heat release rate (HRR)

**Fig. 2:**
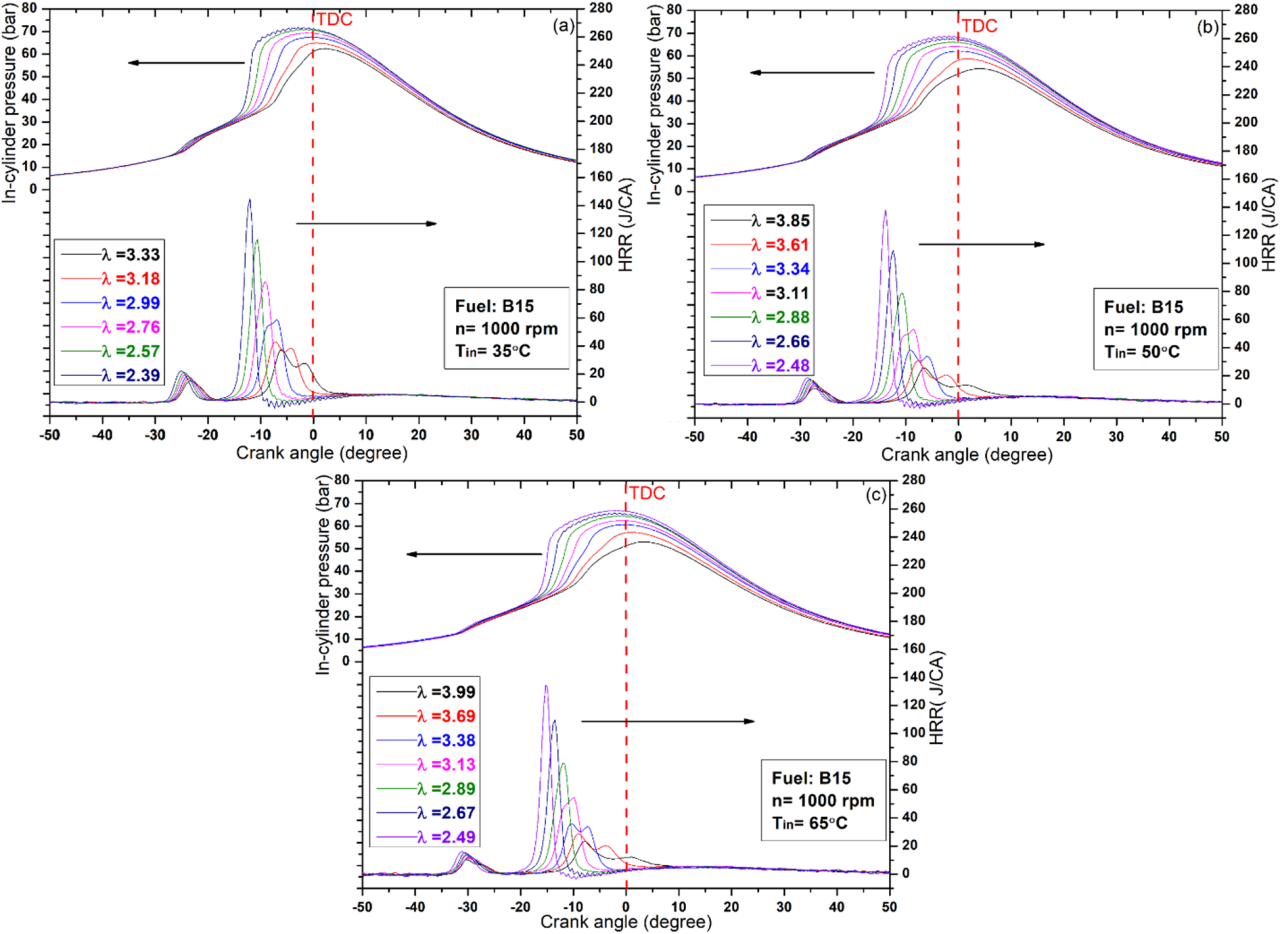
HRR and in‑cylinder pressure of the B15 fuel blend across varying IAT and λ conditions.

**Fig. 3:**
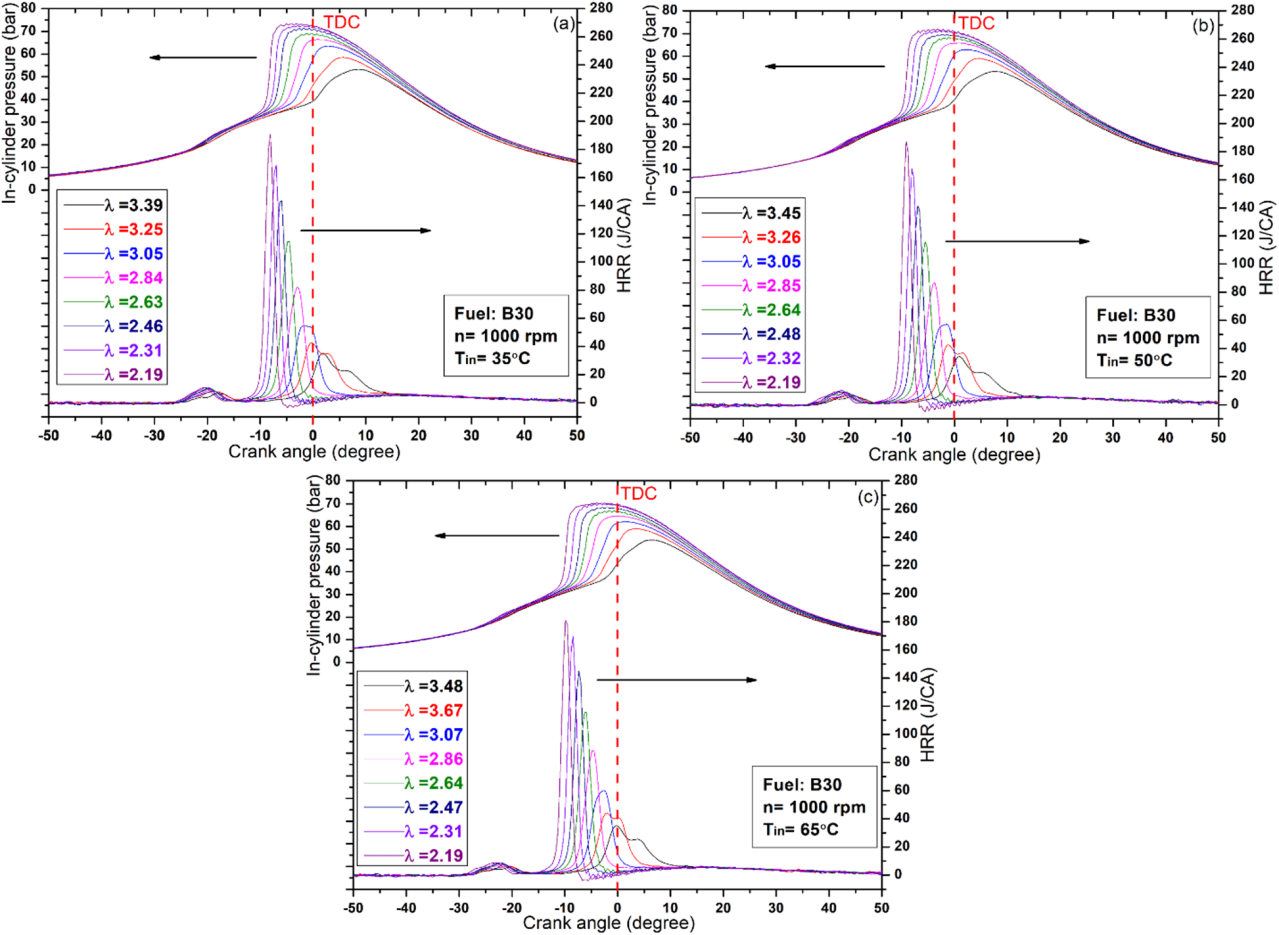
HRR and in‑cylinder pressure of the B30 fuel blend across varying IAT and λ conditions.

**Fig. 4:**
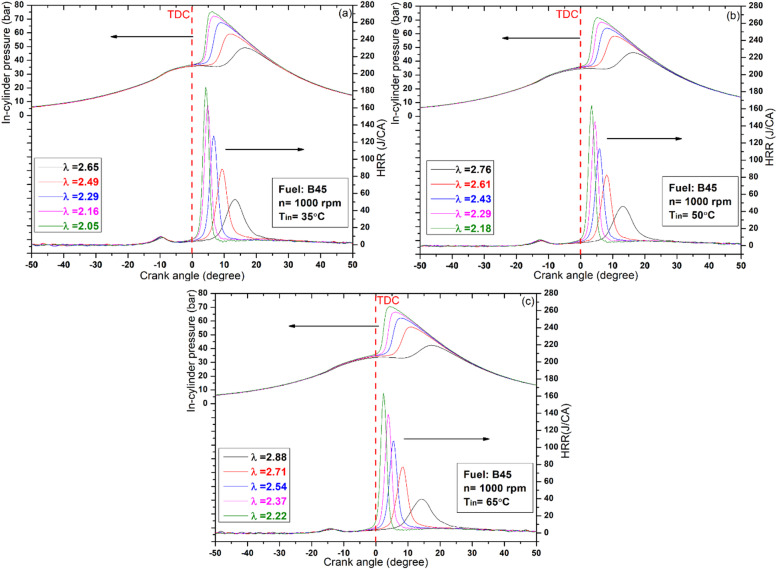
HRR and in‑cylinder pressure of the B45 fuel blend across varying IAT and λ conditions.

Figures 2 and 3, and 4 illustrate the variations in cylinder pressure and HRR for all test fuels across different IATs and lambda values. Throughout the experiments, the engine speed and CR were held constant at 1000 rpm and 12, respectively. For comparative accuracy, both in‑cylinder pressure and HRR data were referenced to the same crank‑angle scale. The HRR results confirm that all test fuels exhibited a distinct two‑stage heat‑release pattern, serving as a reliable indicator of complete HCCI combustion across the investigated IATs. The initial minor peak corresponds to the low‑temperature heat release, typically occurring at 600–800 K, while the subsequent peak corresponds to the high‑temperature heat release phase. Between these two stages lies the negative temperature coefficient (NTC) region, whose characteristics are strongly dependent on fuel reactivity^38^. As illustrated in Figs. 2 and 3, increasing the proportion of high‑reactivity fuels (DEE) consistently produced a two‑stage heat‑release profile. In contrast, higher fractions of low‑reactivity fuels (butanol) yielded a single‑stage pattern, as shown in Fig. 4^39^. This behavior is primarily due to the longer evaporation time and slower homogenization of the butanol mixture, combined with its higher ignition resistance, which is associated with an elevated octane number. Consequently, the typical two‑stage combustion process in HCCI operation tends to converge toward a single‑stage profile when low‑reactivity fuels are employed^40^. Consistent with these observations, the B45 blend exhibited the lowest peak levels of low-temperature oxidation across all IAT conditions investigated. In the high‑temperature heat‑release stage, increasing the DEE fraction in the blends, particularly in B15 and B30, improved the feasibility of HCCI combustion with leaner mixtures. This increase also advanced combustion timing, allowing the process to complete before TDC. This outcome is attributed to DEE’s function as an ignition improver, whereby its oxygenated structure, low octane rating, and low spontaneous ignition temperature collectively accelerate the onset of oxidation reactions through earlier fuel/air interactions^41^. Conversely, increasing the butanol fraction to 45%, as shown in Fig. 4, prevented the achievement of HCCI combustion with leaner mixtures. This outcome is mainly due to butanol’s higher octane number, which delays combustion beyond TDC. As a result, most of the heat release shifts into the expansion stroke, leading to a rapid pressure drop as the cylinder volume increases. In addition, the greater latent heat of vaporization, together with its higher density and viscosity, slows fuel evaporation and impedes mixture homogenization, ultimately postponing the onset of combustion. A comparable investigation by Li et al^42^. reported that increasing the butanol proportion in the fuel blends delayed the combustion phasing, thereby shifting the peak HRR to after TDC.

Building on the HRR analysis, the examination of in-cylinder pressure reveals a similar trend, as shown in the Figures, indicating that peak pressure is sensitive to fuel type, lambda value, and IAT. Hence, increasing the butanol fraction in the blend, as in B45, delays the HCCI combustion phase, as shown in Fig. 4. This delay is consistent with the previously discussed influence of butanol’s fuel properties, which hinder evaporation and mixture preparation relative to DEE. As a result, the combustion phase shifts further into the expansion stroke, where the increasing cylinder volume amplifies heat losses and consequently reduces the peak cylinder pressure. In contrast, the B30 blend produced the highest peak cylinder pressure and HRR values, 73.3 bar and 191 J/°CA, respectively, at an IAT of 35 °C. This behavior becomes clearer when considering that B15 and B45 tend to shift the combustion either noticeably before or well after TDC, whereas B30 maintains the heat release near TDC, thereby achieving the most favorable pressure and temperature conditions for efficient combustion. Additionally, at higher engine loads (i.e., lower λ values), oxidation reactions progressed more rapidly within narrower crank‑angle intervals, which intensified knock tendencies due to the greater fuel energy input, as illustrated in Figs. 2 and 3. On the other hand, increasing λ alleviated these effects, thereby reducing the knocking tendency.

In addition to the effects of λ and fuel composition, IAT also exerts a significant influence on combustion. As demonstrated in Figs. 2, 3 and 4, increasing IAT allows the fuel to reach its auto‑ignition temperature earlier, thereby advancing the combustion phasing. This advancement arises because higher IAT enhances molecular collisions and accelerates radical reaction rates, thereby establishing more favorable initial combustion conditions and offsetting the cooling effect associated with the high latent heat of vaporization in alcohol‑based fuels^43^. Moreover, at higher IATs, HCCI combustion was consistently achieved with leaner mixtures across all tested fuels. As noted earlier, increasing the DEE fraction extended the lean operating limit; however, the effect of elevated IAT proved even more pronounced, further expanding the range of stable HCCI operation. The observed behavior results from the simultaneous elevation of pressure and temperature within the cylinder at the end of compression, thereby promoting earlier auto‑ignition. As a consequence, the combustion process becomes more diluted, reflecting the characteristic influence of elevated IAT on mixture reactivity and ignition phasing. For instance, in the case of the B15 fuel blend, the operating range of lambda values extended from 2.39 to 3.33 at an IAT of 35 °C, from 2.48 to 3.85 at 50 °C, and from 2.49 to 3.99 at 65 °C.

### Indicated mean effective pressure (IMEP)

**Fig. 5:**
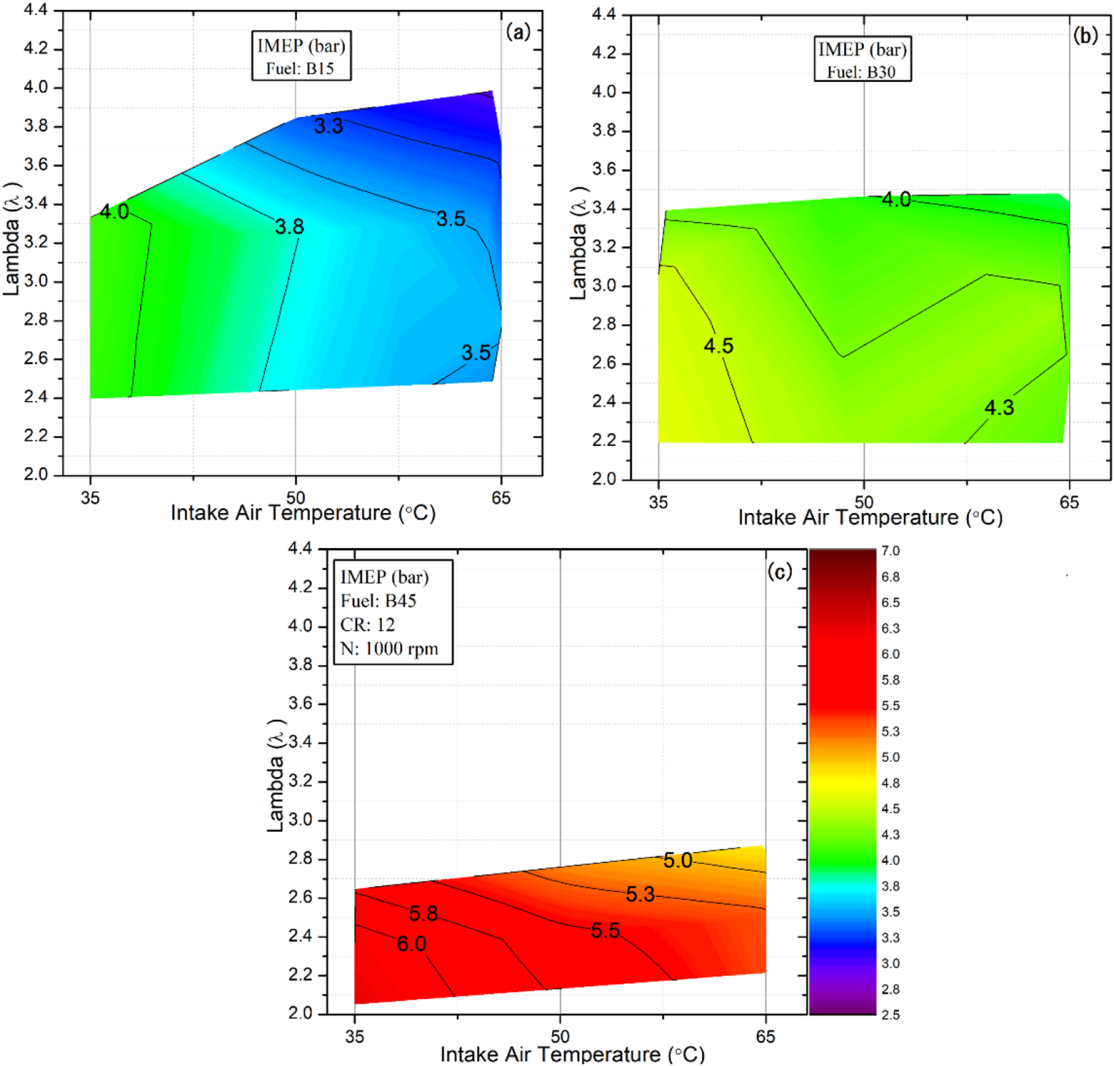
Effect of lambda and IAT on IMEP for different fuel blends.

Figure 5 shows the IMEP HCCI operation maps for all tested fuels under different IAT and λ conditions. The x-axis represents the IAT, while the y-axis corresponds to the λ values. The color contours depict the variation in IMEP as an indicator of engine load. In these maps, the red regions denote the highest engine load, whereas the blue-to-purple regions indicate the lowest-load regime. The same axis arrangement and contour scale are maintained for all corresponding figures in this study to ensure consistency and facilitate comparison.

The results demonstrate that an increase in IAT together with a higher DEE fraction in the blend broadens the operating range of HCCI engines, as illustrated in Fig. 5a. This expansion results from higher charge temperature and fuel reactivity, which shorten ignition delay and advance combustion phasing, enabling stable operation at lower loads. For instance, the operating range of the B15 blend expanded with respect to λ from 3.33 to 3.848 and 3.992 as the IAT increased from 35 °C to 50 °C and 65 °C, respectively. However, in both cases, expanding the operating range is accompanied by a gradual reduction in IMEP. As IAT increases and DEE fractions rise, combustion occurs earlier, thereby increasing negative work and leading to a decline in IMEP^43^. Since IMEP depends on net indicated work over the swept volume, early heat release before TDC significantly reduces the effective pressure output. For example, the IMEP of the B30 blend decreased from 4.65 to 4.44 and 4.29 bar as the IAT increased from 35 °C to 50 °C and 65 °C, respectively.

On the other hand, increasing the butanol content from 30% to 45% in the fuel blends delayed combustion onset due to its lower reactivity. shiftting combustion closer to the optimal crank angle, as shown in Fig. 5b and c. This adjustment improved the thermodynamic conditions during expansion, resulting in a better pressure profile, improved thermal efficiency, and increased IMEP. For instance, at 35 °C, the IMEP for the B45 fuel increased by 46% and 29% relative to the B15 and B30 blends, respectively. Moreover, as shown in Fig. 5c, increasing the butanol content in the blend shifts HCCI combustion toward richer mixture regions. Hence, stable HCCI combustion cannot be maintained under excessively lean conditions. Fuels with lower chemical reactivity, in particular, require higher IAT to achieve reliable operation. This behavior results from the higher butanol fraction, which slows evaporation, delays mixture formation, and raises the blend’s octane number. In addition, IMEP increases at lower λ values due to greater energy released into the cylinder, whereas at higher λ values it decreases accordingly. Consequently, the highest IMEP value of 6.27 bar for the B45 blend was recorded at an IAT of 35 °C and a λ value of 2.05, corresponding to the richest operating region. Overall, B15 exhibits the widest stable λ range due to higher DEE-induced reactivity, while B45 achieves the highest IMEP owing to delayed combustion and optimal phasing. Increasing IAT advances combustion, raising negative work and reducing IMEP, as early heat release before TDC limits the effective pressure output. These results highlight the combined effect of fuel composition and IAT on HCCI performance and stability.

### The start of combustion (CA10)

**Fig. 6:**
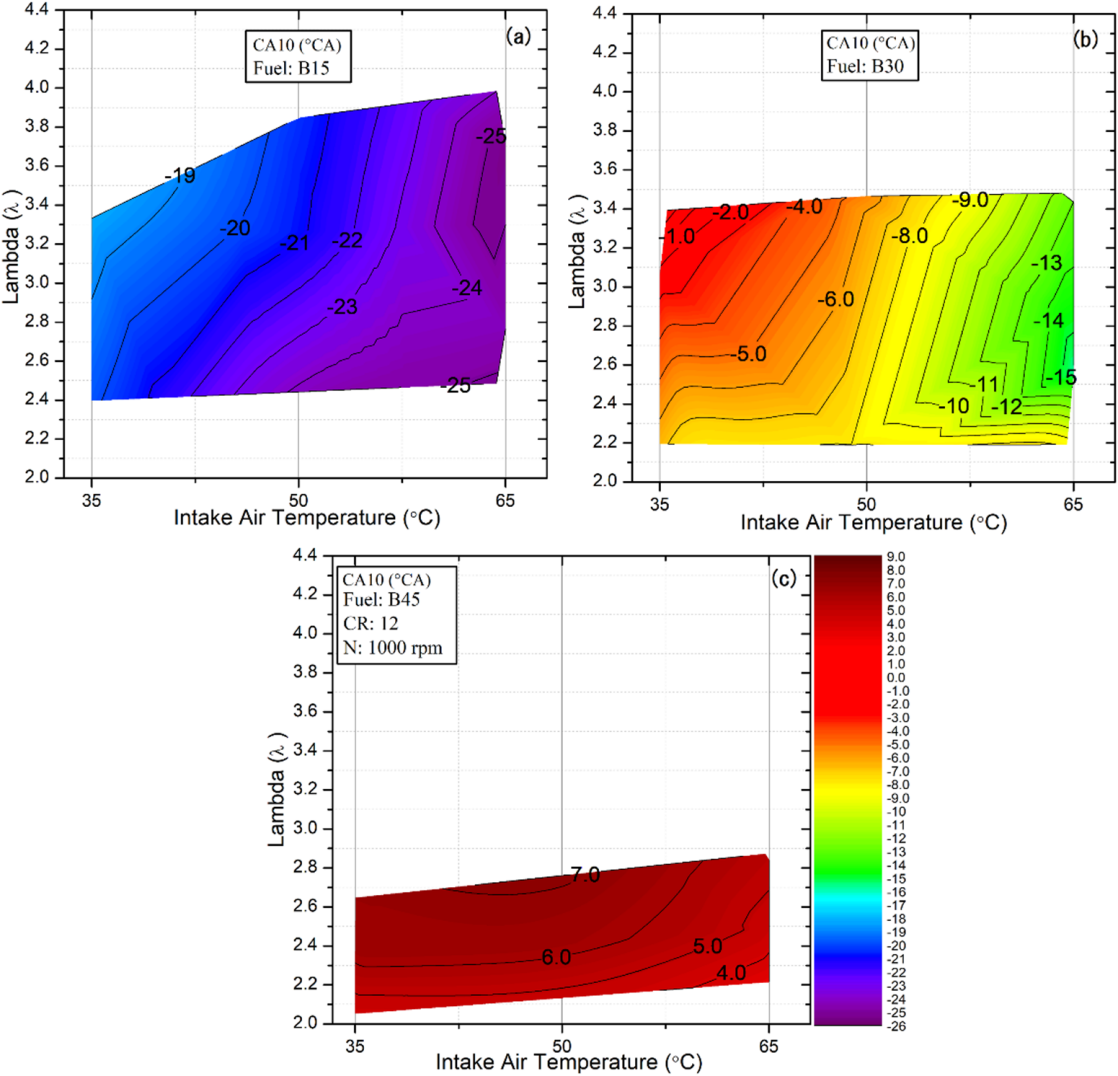
Effect of lambda and IAT on CA10 for different fuel blends.

The start of combustion, commonly referred to as CA10 or SOC in HCCI engine research, is defined as the crank angle at which 10% of the fuel/air mixture has been burned. Because HCCI engines lack a conventional mechanism to control SOC directly, it is primarily determined by mixture composition and the cylinder’s thermal history. Consequently, parameters such as IAT, λ, boost pressure, and engine speed are critical for controlling the initiation of combustion^44^. Figure 6 illustrates the combined influence of IAT, λ, and fuel type on CA10 under constant engine speed and compression ratio. Figure 6a shows that CA10 occurs earlier across different IAT and lambda values for the B15 fuel blend. This advancement stems from the inherent characteristics of DEE, namely its high cetane rating and low auto‑ignition temperature, enabling combustion to commence before TDC. Whereas, increasing the butanol fraction in the blend from 30% to 45%, as illustrated in Fig. 6b and c, results in a clear retardation of CA10 at all IATs. This shift is primarily due to butanol’s lower cetane number, which increases its autoignition resistance and reduces the likelihood of early ignition. Moreover, the higher latent heat of vaporization of n-butanol than that of DEE requires additional energy input during the intake and compression strokes to achieve complete vaporization. As a result, the mixture temperature decreases, delaying the onset of CA10. This trend is evident, for example, at an IAT of 65 °C and a lambda value of 2.7, where combustion with the B45 blend begins only 5.04° after TDC. Under the same conditions, the ignition onset occurs much earlier with the B15 and B30 blends, at 24.2° and 13.68° before TDC, respectively. Compared with B15 blends, alcohol-rich B45 blends with higher octane numbers exhibit delayed ignition, which suppresses abnormal combustion, particularly knocking, and extends the stable operating range into higher-load conditions, making them more suitable for HCCI applications.

Building on the previously discussed influence of fuel composition on CA10 timing, IAT introduces an additional layer of sensitivity to combustion phasing across all blends. When examining the impact of IAT on the different fuel types, it becomes evident that higher IAT results in greater CA10 advance, as illustrated in Fig. 6. This advancement stems from improved mixture preparation and the greater number of thermally activated molecules, both of which accelerate fuel-cracking and oxidation reactions and thereby increase the overall reaction rate. For example, with the B30 blend at a lambda value of 2.5, CA10 advanced markedly from 3.96° to 8.64° and 13.92° BTDC as the IAT increased from 35 °C to 50 °C and 65 °C, respectively. Cinar et al^45^. reported that increasing the IAT accelerates combustion by triggering earlier chemical reactions between oxygen and hydrocarbon molecules, underscoring the crucial role of intake conditions in controlling auto-ignition behavior. A similar trend is observed when the mixture becomes richer: the greater fuel quantity in the cylinder increases the probability of fuel–oxygen interactions, promoting earlier initiation of oxidation reactions and thus advancing CA10 for all blends. Conversely, under leaner mixture conditions, reduced fuel availability limits these interactions between hydrocarbon and oxygen molecules, delaying the onset of combustion.

### Indicated thermal efficiency and CA50

**Fig. 7:**
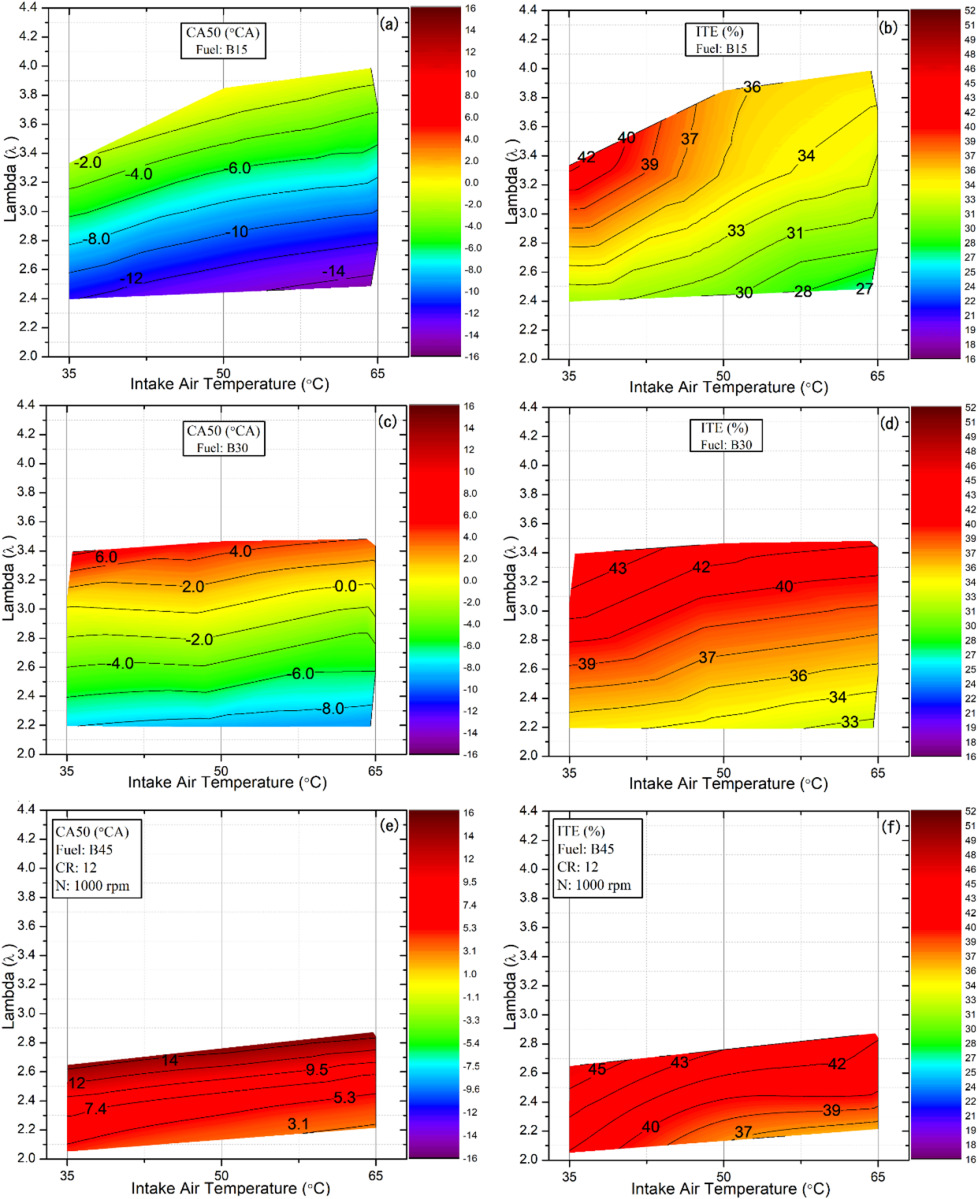
Effect of lambda and IAT on CA50 and ITE for different fuel blends.

Indicated thermal efficiency (ITE) is a fundamental metric for quantifying how much of the fuel’s chemical energy is converted into net mechanical output. It reflects the overall effectiveness of the engine’s energy conversion^46^. CA50, on the other hand, represents the crank angle at which 50% of the total fuel mass has been burned within the cylinder^47^. The timing of CA50 is critical in determining the ITE of internal combustion engines; therefore, both parameters are presented together to facilitate better correlation and analysis. Achieving a CA50 of 7°–11° after TDC is generally associated with optimal combustion phasing. Leading to improved engine performance and higher ITE in practical internal combustion engine applications^48^. Advancing CA50 beyond this range increases the likelihood of knock and results in greater negative work on the piston. In contrast, retarding CA50 further into the expansion stroke increases heat losses. In both cases, deviations from the optimal CA50 range in either direction lead to a decrease in the engine’s thermal efficiency^49,50^. Figure 7 shows the effect of fuel blends, IAT, and λ on CA50 and ITE in HCCI mode, with CA50 values on the right axis and ITE on the opposite axis.

Figure 7 (a, c, e) indicates that CA50, similar to CA10, advanced as λ decreased across all fuels. This advancement is attributed to elevated in‑cylinder temperatures in rich mixtures, which accelerate chemical reactions. Under these conditions, more fuel is needed to maintain the same power, increasing fuel consumption and lowering thermal efficiency (Fig. 7b, d, f). In contrast, increasing the proportion of butanol in the fuel blend enhances autoignition resistance. This is due to its higher octane rating and greater latent heat of vaporization, which together lower in-cylinder temperature during compression. Consequently, the CA50 shifts beyond TDC, improving the ITE. This observation aligns with Heywood’s finding that optimal thermal efficiency is achieved when CA50 occurs slightly after TDC^51^. Moreover, the reduction in MPRR at higher butanol ratios indicates smoother combustion behavior, further supporting the overall efficiency enhancement. For example, the CA50 values for the B15 and B30 blends were 11.34°CA and 5.31°CA before TDC, respectively, whereas for the B45 blend, CA50 was significantly delayed to 9.72°CA after TDC. Retarding the combustion phasing with B45 within the optimal range improved the alignment of heat release with the expansion stroke, maximizing energy conversion efficiency. As a result, the ITE of the B45 blend increased by 20% and 8.24% compared with B15 and B30, respectively.

A comprehensive analysis of Fig. 7 reveals that increasing the IAT results in a decrease in the ITE, as the CA50 shifts to earlier crank angles across all tested fuels. This temperature rise accelerated the fuel–air reaction rate, enhancing both vaporization and oxidation processes and causing combustion to occur earlier in the cycle. However, excessively high intake temperatures can reduce engine efficiency by advancing combustion, causing combustion instability and lower indicated power. For the B45 blend, the CA50 values, which initially fell within the optimal range of 7–11°CA at 35 and 50 °C, shifted outside this range at an IAT of 65 °C. Additionally, increasing the IAT reduces the intake‑air density, thereby lowering the mass of air inducted into the cylinder. This deterioration in the mixture’s preparation reduces combustion quality and diminishes overall efficiency. Under these conditions, the B45 fuel blend achieved a maximum ITE of 45.78% at a lambda value of 2.64 and an IAT of 35 °C.

### The combustion duration (CD)

**Fig. 8:**
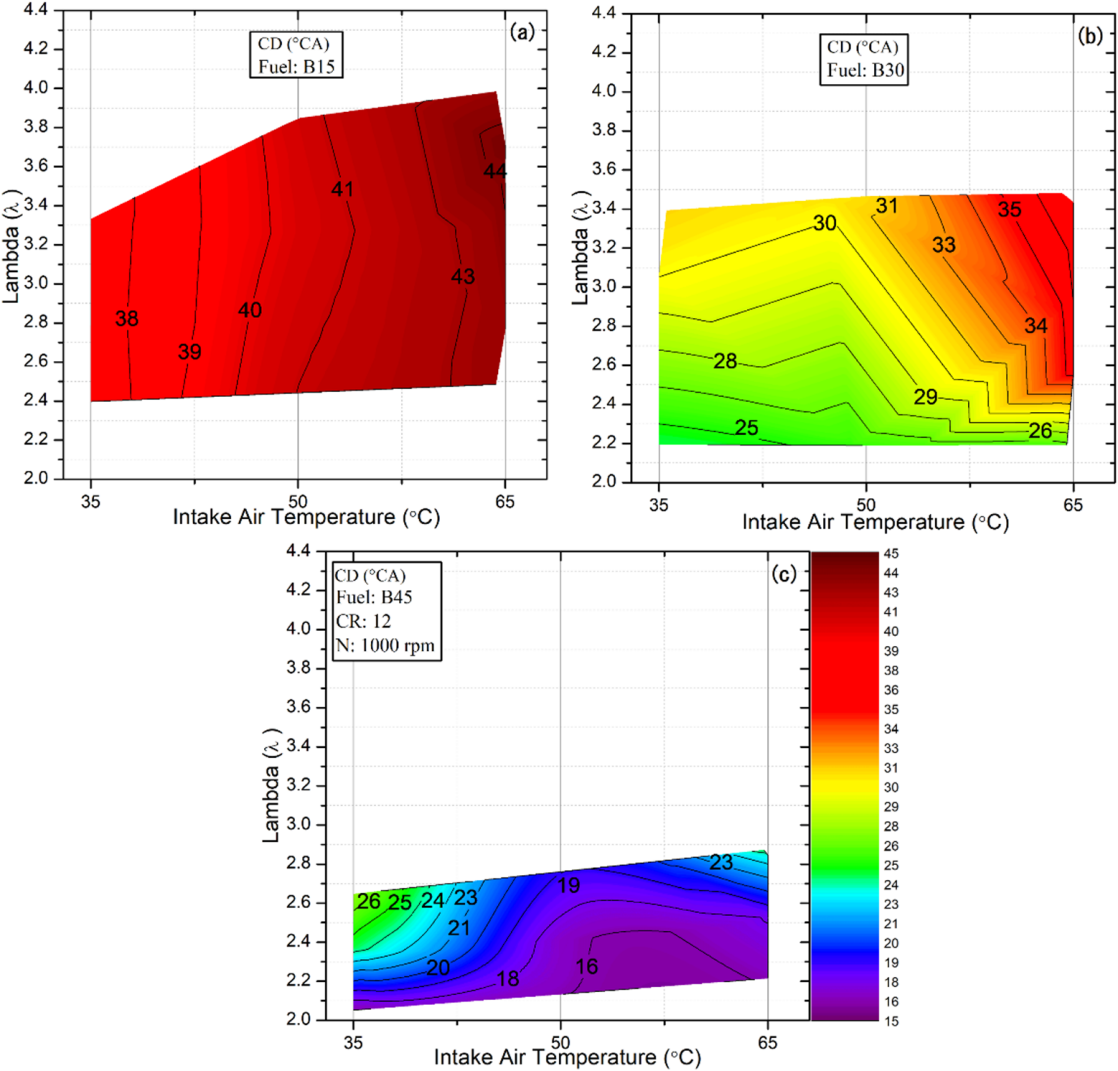
Effect of lambda and IAT on combustion duration for different fuel blends.

It is another key parameter that strongly affects fuel conversion efficiency in HCCI engines. Determining the precise end of combustion, however, presents a significant challenge in this combustion mode because a portion of the heat release is lost to the cylinder walls and some fuel undergoes incomplete oxidation. These factors obscure the exact point at which combustion ceases. For this reason, the CA10–90 interval is widely used as a reliable indicator of the overall combustion duration, providing a consistent and practical basis for comparing different operating conditions and fuel blends^52^. Figure 8 demonstrates the CD of the tested fuels under different IAT and λ values. Across all fuels and temperatures, a clear trend emerges: combustion takes longer to complete as the mixture becomes leaner. This extended duration is mainly due to the slower chemical kinetics in fuel‑deficient mixtures^53^. Consequently, both the onset and completion of combustion shift to later crank angles, reflecting the diminished reactivity of lean mixtures. In addition to the influence of mixture strength, fuel composition further clarifies the variations in combustion duration across the tested conditions. The findings reveal that the CD decreases with increasing butanol concentration in the fuel blend, reaching a minimum at a 45% butanol ratio. Although higher butanol content tends to delay the onset of combustion, as reflected in a later CA10 (Fig. 6), this does not translate into a longer CD. Instead, once ignition is established, the elevated butanol fraction promotes a more rapid, uniform combustion. The accelerated combustion is primarily attributed to increased hydroxyl radical generation, which promotes reactions within the highly diluted mixture and consequently enhances the overall burning rate of the blends. The current observation is consistent with the results of He et al^48^., who reported a similar trend. Consequently, the B45 blend consistently exhibited the shortest CD, whereas the B15 blend showed the longest duration due to its greater propensity to knock. For example, at an IAT of 50 °C and a lambda value of 2.5, the CD for B45 was reduced by 59% and 38% relative to B15 and B30, respectively.

On the other hand, when assessing the influence of IAT on combustion behavior, the literature is well-established that elevated charge temperatures generally lead to shorter CD values, owing to enhanced air/fuel chemical activity and a greater number of energetically activated molecules that accelerate oxidation reactions^54^. These effects typically promote faster heat release and, consequently, a reduced overall combustion duration. However, the present study revealed the opposite trend: increasing the IAT across all tested fuel blends resulted in a longer combustion duration. This behavior is primarily explained by the observation that higher IAT values advance combustion phasing, shifting a substantial portion of the heat‑release process into the expansion stroke. As combustion proceeds during expansion, the larger cylinder volume lowers the in‑cylinder gas temperature and slows the overall reaction rate. At the same time, the increased volume intensifies heat transfer to the cylinder walls and to the exhaust gases, thereby increasing thermal losses. These combined effects reduce combustion efficiency and prolong the time required for the process to reach completion compared with lower IAT conditions. For instance, for the B30 fuel blend at a lambda value of 2.2, the combustion duration increased progressively from 24.3°CA to 25.38°CA and 26.1°CA as the intake air temperature rose from 35 °C to 50 °C and 65 °C, respectively. Similar findings were reported by Hasan et al^55^., who observed that increasing the IAT prolonged the combustion duration due to faster auto‑ignition. Accordingly, increasing the octane number of the B45 fuel blend shortens the CD, contrary to the usual trend. In contrast, a higher cetane number in B15, along with elevated temperatures, extends the CD; nevertheless, the effects of fuel ratio and lambda values on combustion duration are more significant than those of the IAT.

.

### The maximum pressure rise rate (PRR_max_)

**Fig. 9:**
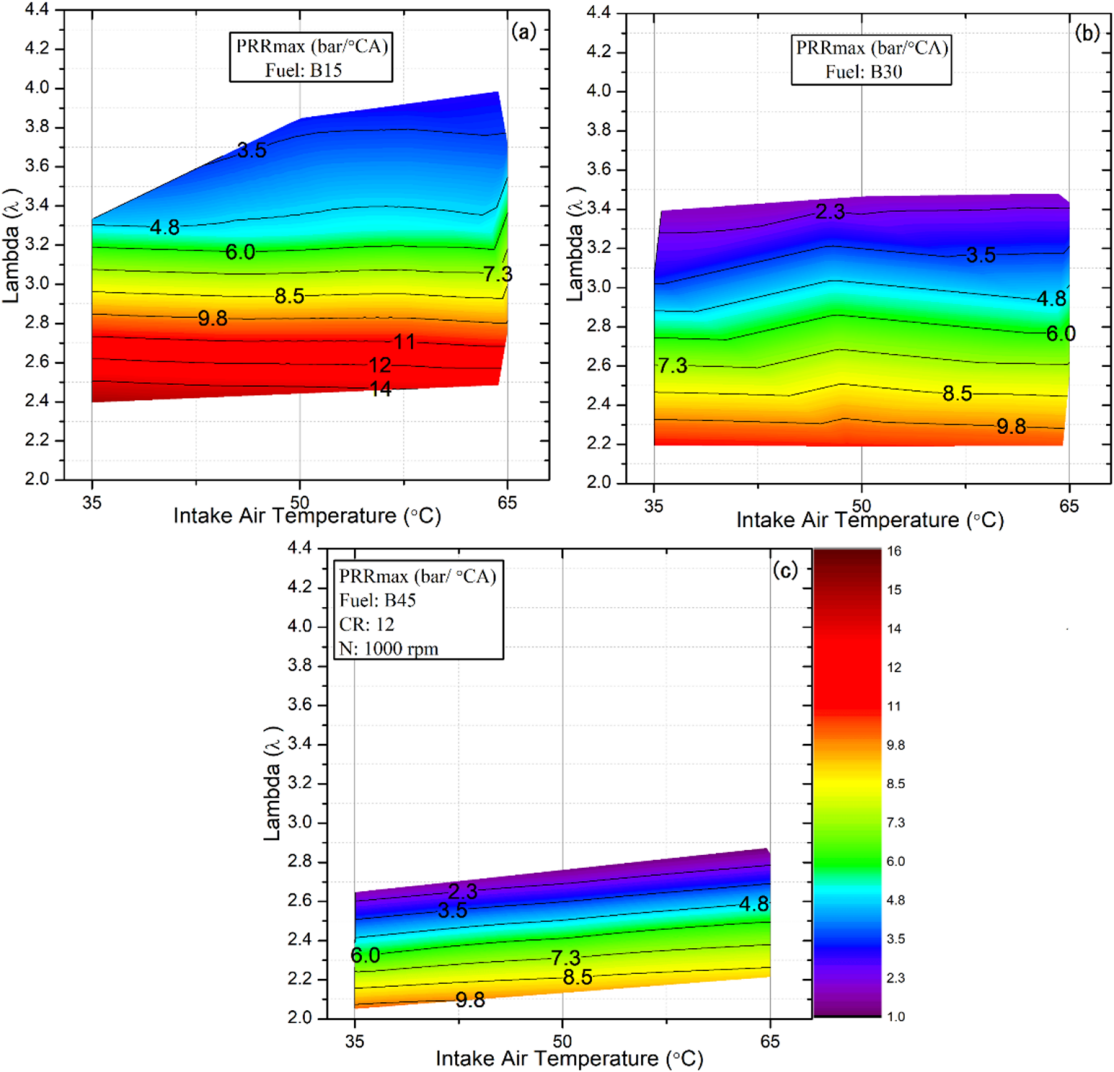
Effect of lambda and IAT on PRR_max_ for different fuel blends.

A sharp increase in heat release over a very narrow crank‑angle range leads to a rapid pressure rise and intensified knocking, a key factor constraining the operational range of HCCI engines. When the PRR_max_ exceeds the critical threshold of 10 bar/°CA, excessive knocking occurs, and stable HCCI combustion can no longer be sustained^56,57^. Figure 9 illustrates the variation in PRRmax across fuel blends at lambda values and IATs of 35, 50, and 65 °C.

The results indicate that increasing the DEE fraction in the B15 blend at lower λ values accelerates combustion and significantly increases PRRₘₐₓ. As illustrated by the red regions in Fig. 9a, PRRₘₐₓ exceeds the knock-limited threshold, reaching a maximum value of 14 bar/°CA at λ = 2.39, thereby confirming the onset of severe knocking compared with the other test fuels. This behavior is primarily attributed to the intrinsic properties of DEE, namely its high cetane number and low autoignition temperature, as well as the larger fuel quantity introduced into the cylinder at lower λ values. The combustion of this increased fuel mass within a very narrow crank-angle interval produces an extremely rapid heat release, leading to a sharp temperature rise and a steep increase in in-cylinder pressure^58^. Consequently, reducing λ below a critical threshold is not feasible in HCCI operation, as the associated high pressure-rise rates induce excessive noise, strong vibrations, and unstable engine behavior. Despite the early occurrence of peak pressure shown in Fig. 2 and the tendency toward knock under rich conditions, B15 maintained stable combustion over a relatively wider λ range than the other tested blends. In HCCI combustion, the effective operating range is determined by the stability limits imposed by knock (high PRRₘₐₓ) under rich conditions and by combustion instability or misfire under lean conditions. Accordingly, the λ values presented in Fig. 9 represent the corresponding engine load range. For the B15 blend, the onset of knock at λ = 2.39 defines the rich-side operating limit, whereas stable combustion is maintained up to approximately λ ≈ 4.0, resulting in the widest stable λ operating range among the tested blends. Compared with DEE’s low knock resistance, increasing the butanol fraction from 30% to 45% improved the blend’s knock resistance. This enhancement enabled the engine to operate under conditions farther removed from the knock threshold, as demonstrated in Fig. 9b and c. This decrease can be attributed to butanol’s properties in the fuel mixture. One factor is the cooling effect from its high latent heat of vaporization, which reduces the in-cylinder temperature at the end of the compression stroke. Another factor is its high octane rating, which extends combustion over a wider crank angle range, thereby slowing combustion. Both effects ultimately result in a reduction of PRR_max_^48^. Moreover, the incorporation of n‑butanol into DEE shifted the HCCI operating range toward higher engine loads. Consequently, the B30 blend maintained a λ of approximately 2.19 across all IATs, whereas increasing the butanol fraction to B45 further extended the attainable load range, reducing λ to approximately 2.05–2.20. These results emphasize that, compared with B15, the B45 blend highlights the importance of higher octane to sustain HCCI combustion under elevated-load conditions. Without sufficient octane resistance, stable HCCI operation cannot be maintained, and the engine shuts down once the load exceeds this threshold. For instance, the PRRmax value decreased from 13.7 bar/CA for the B15 blend to 4 bar/CA for the B45 blend at an intake air temperature of 35 °C and a lambda value of 2.5.

While fuel octane and composition clearly dominate the combustion stability, previous studies have reported that high IAT has a direct effect on oxidation reactions, accelerating reaction rates and intensifying knocking^59^. The present results demonstrate that the influence of IAT on knocking tendency is relatively minor compared with the dominant effects of fuel composition and λ, as shown in Fig. 9. Increasing the IAT advances CA10 further away from TDC, as illustrated in Fig. 6, thereby reducing the HRR and peak in‑cylinder pressure during the later stages of combustion. This reduction mitigates the tendency for knock despite the higher charge temperature. For instance, under a λ value of 2.3 with the B30 blend, PRRmax decreased slightly from 9.87 to 9.73 and 9.72 bar/°CA as IAT increased from 35 °C to 50 °C and 65 °C, respectively.

### The coefficient of variation (COV_IMEP_)

**Fig. 10:**
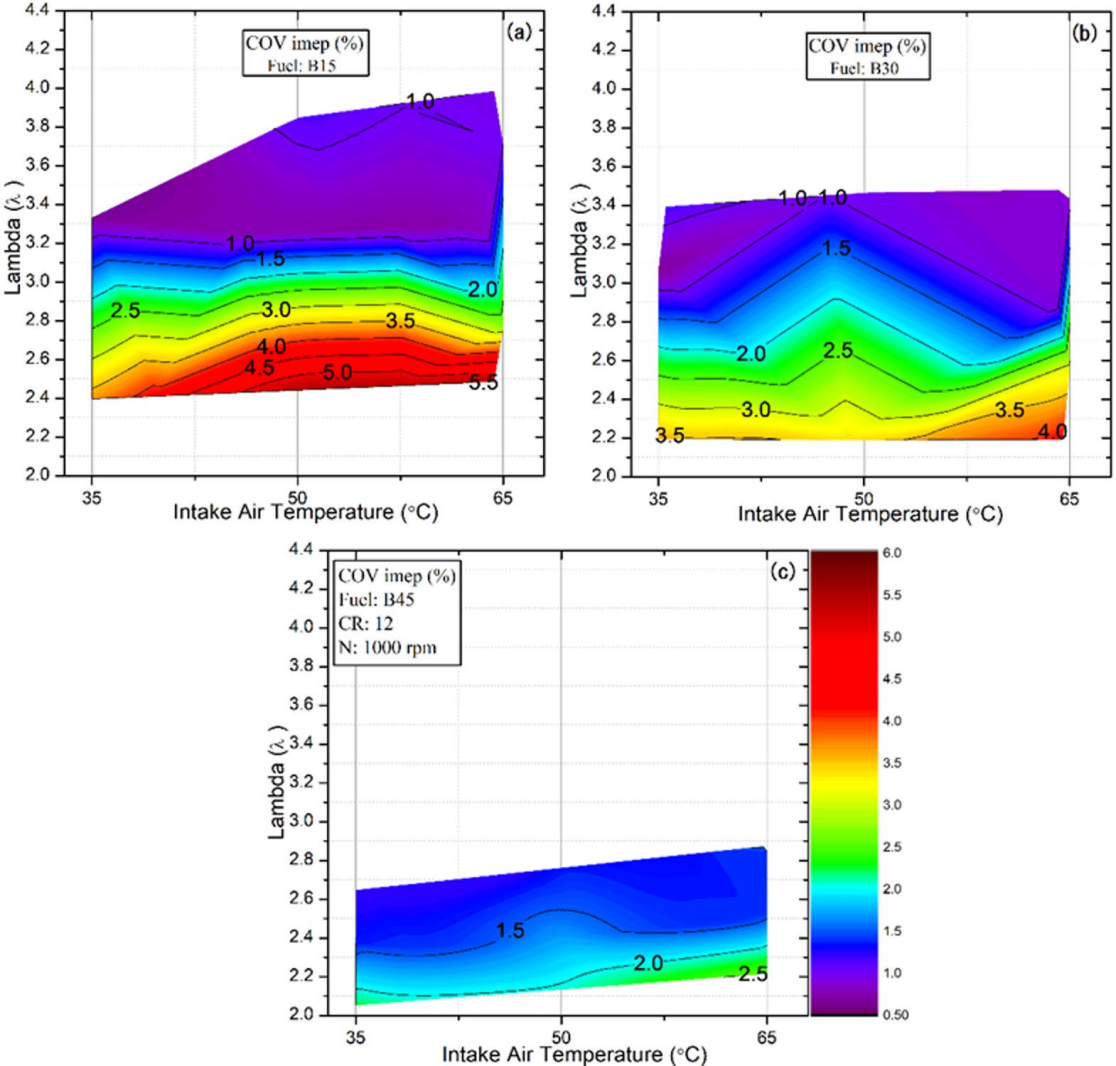
Effect of lambda and IAT on COV_IMEP_ for different fuel blends.

One of the principal limitations of HCCI engines is their inherently narrow operating range, since knocking tends to occur at high loads while misfiring becomes problematic at low loads. These adverse conditions intensify cycle‑to‑cycle variations, resulting in unstable engine speed, noticeable fluctuations, and, in severe cases, complete stalling^57,60^. Cyclic variability is typically quantified using the COV_IMEP_ to accurately assess combustion quality and operational stability under varying loads and operating conditions^61,62^. Earlier research has suggested that keeping COV_IMEP_ below 10% is essential for stable engine performance^63^. Figure 10 illustrates the variation of COV_IMEP_ for the tested fuel blends under different IAT and λ conditions. The results indicate that COV_IMEP_ ​generally decreases with increasing λ and higher butanol content in the fuel blend across all IAT conditions. Increasing λ reduces the in-cylinder peak pressure and heat-release rate, thereby suppressing knock across all tested fuels. This behavior is mainly associated with delayed combustion phasing under leaner mixture conditions, where a smaller amount of fuel is inducted and burned in the combustion chamber, leading to reduced cycle-to-cycle variations^64^. For example, for the B15 blend at an IAT of 35 °C, increasing λ from 2.4 to 2.6 and 2.8 reduced the COV_IMEP_ from 3.6% to 2.8% and 2.01%, respectively. Building on this trend, increasing the butanol content in the fuel blend (from B15 to B45) further reduced the knocking tendency, as shown in Fig. 9b and c, owing to its higher octane number and auto-ignition temperature than DEE. This enhanced knock resistance resulted in more stable HCCI combustion, as evidenced by lower COV_IMEP_ values. For instance, at an IAT of 35 °C and a λ of 2.4, COV_IMEP_ ​ decreased from 3.7% for B15 to 3.1% and 1.22% for the B30 and B45 blends, respectively. This observation is consistent with Aydoğan’s findings, who reported that increasing the fuel blend’s octane number effectively reduces cycle-to-cycle variability^65^. On the other hand, while higher butanol fractions reduce cyclic variability at a given λ, elevated IAT deteriorates combustion quality, limits controllability, and increases cycle variability. A similar trend was reported by Rojas et al^66^., observed that increasing the IAT led to higher COV_IMEP_ ​values. Consequently, at an IAT of 65 °C, the COV_IMEP_ ​​ for the B15 blend increased by 54% and 15% compared with the corresponding values at 35 °C and 50 °C, respectively. Nevertheless, despite the adverse influence of higher IAT on the combustion quality, all recorded COV_IMEP_ ​​ values remained below 10%, indicating that stable operation was maintained within the admissible range for the test engines.

### Emissions of HC

**Fig. 11:**
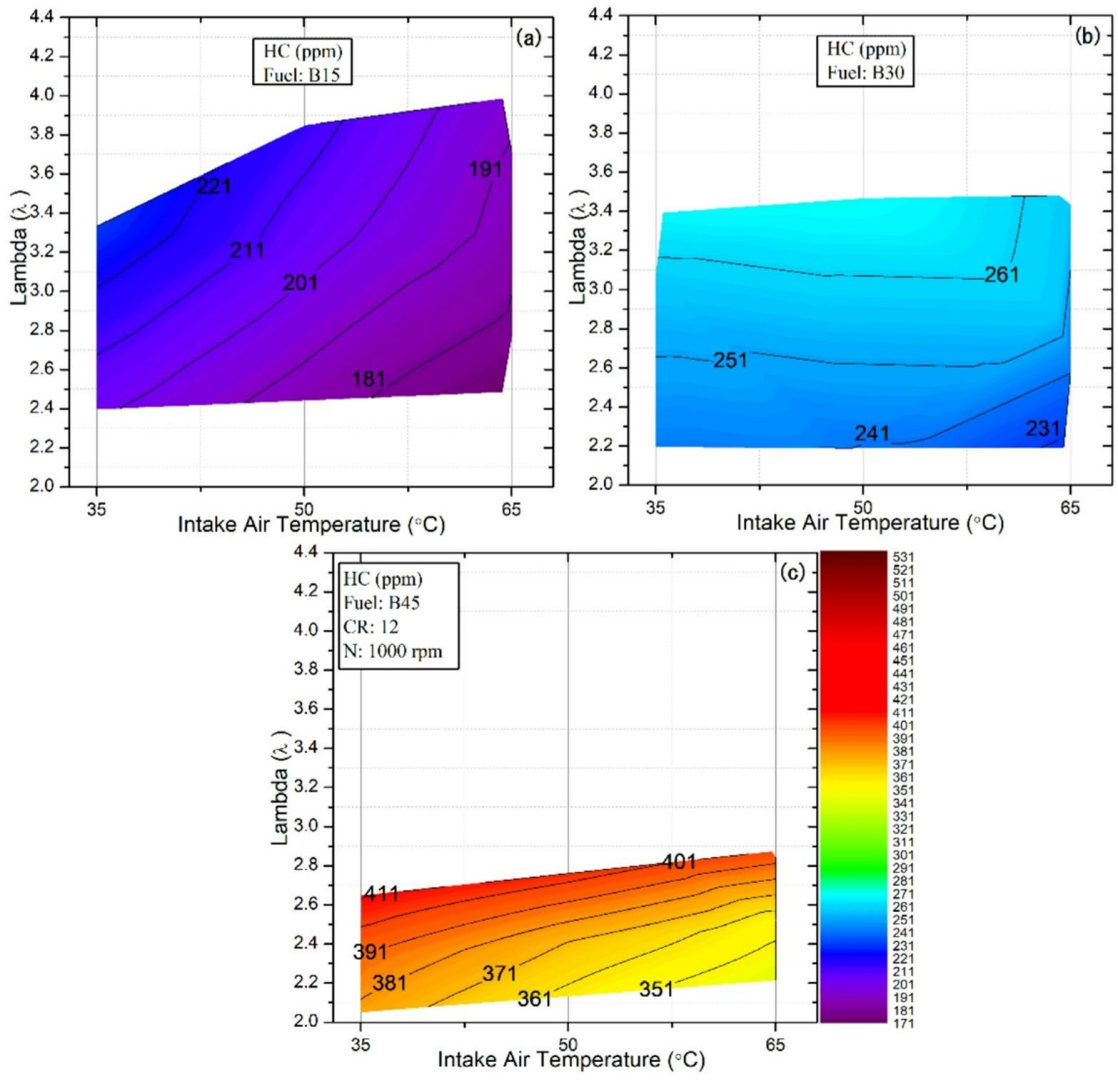
Effect of lambda and IAT on HC emissions for different fuel blends.

HCCI combustion typically results in elevated HC and CO emissions relative to conventional spark-ignition (SI) and compression-ignition (CI) engines, primarily due to its low-temperature, lean-burn nature and the absence of a controlled flame front, which can lead to incomplete oxidation of fuel species. Specifically, HC emissions are often attributed to incomplete combustion in localized regions, particularly where unburned fuel becomes trapped, such as in crevice volumes, and where heat losses near the cylinder walls suppress reaction rates, further impeding complete oxidation of the fuel^67,68^. Figure 11 illustrates the influence of test fuels under different IAT and λ values on HC emissions in HCCI mode. The results clearly demonstrate that HC emissions consistently decrease with increasing IAT across all fuels and operating conditions. This reduction is attributed to enhanced chemical reactions and rapid combustion at elevated IAT, which accelerate radical formation and intensify combustion, thereby reducing the cooling effect of the lean homogeneous mixture^69,70^. Beyond the thermal effect, increasing the DEE concentration in fuel blends further reduces HC emissions. This enhancement is attributed to the high oxygen content of DEE and its low carbon-to-hydrogen ratio, which promotes cleaner combustion chemistry. Furthermore, the low density and viscosity of DEE enhance fuel atomization and air/fuel mixing, thereby facilitating more efficient oxidation reactions and reducing the prevalence of unburned species^71^. This observation is consistent with previously reported findings on DEE^41^. This dual effect highlights the importance of optimizing both operating conditions and fuel formulation to achieve substantial reductions in HC emissions under the HCCI model. Conversely, increasing the butanol proportion in the fuel blends led to a marked increase in HC emissions, with the B45 blend exhibiting the highest levels. This escalation is primarily attributed to butanol’s higher latent heat of vaporization and lower cetane number, which collectively hinder fuel evaporation and reduce chemical reactivity. As a result, ignition is delayed, and combustion duration is shortened, as illustrated in Fig. 8c, leading to incomplete combustion and elevated emissions^72^. The combined influence of IAT and fuel composition on HC emissions is clearly illustrated by the present results. The lowest emissions were observed for the B15 blend at an IAT of 65 °C, with 171 ppm, whereas the highest emissions were observed for the B45 blend at 35 °C, with 203 ppm. This behavior can be attributed to the higher DEE content in B15 and the elevated IAT, which together reduce HC formation, whereas the elevated butanol fraction in B45 has an adverse effect. Similarly, increasing λ reduces the fuel energy delivered to the cylinder, thereby lowering the post‑combustion gas temperature and, consequently, leading to higher HC emissions.

### Emissions of CO

**Fig. 12:**
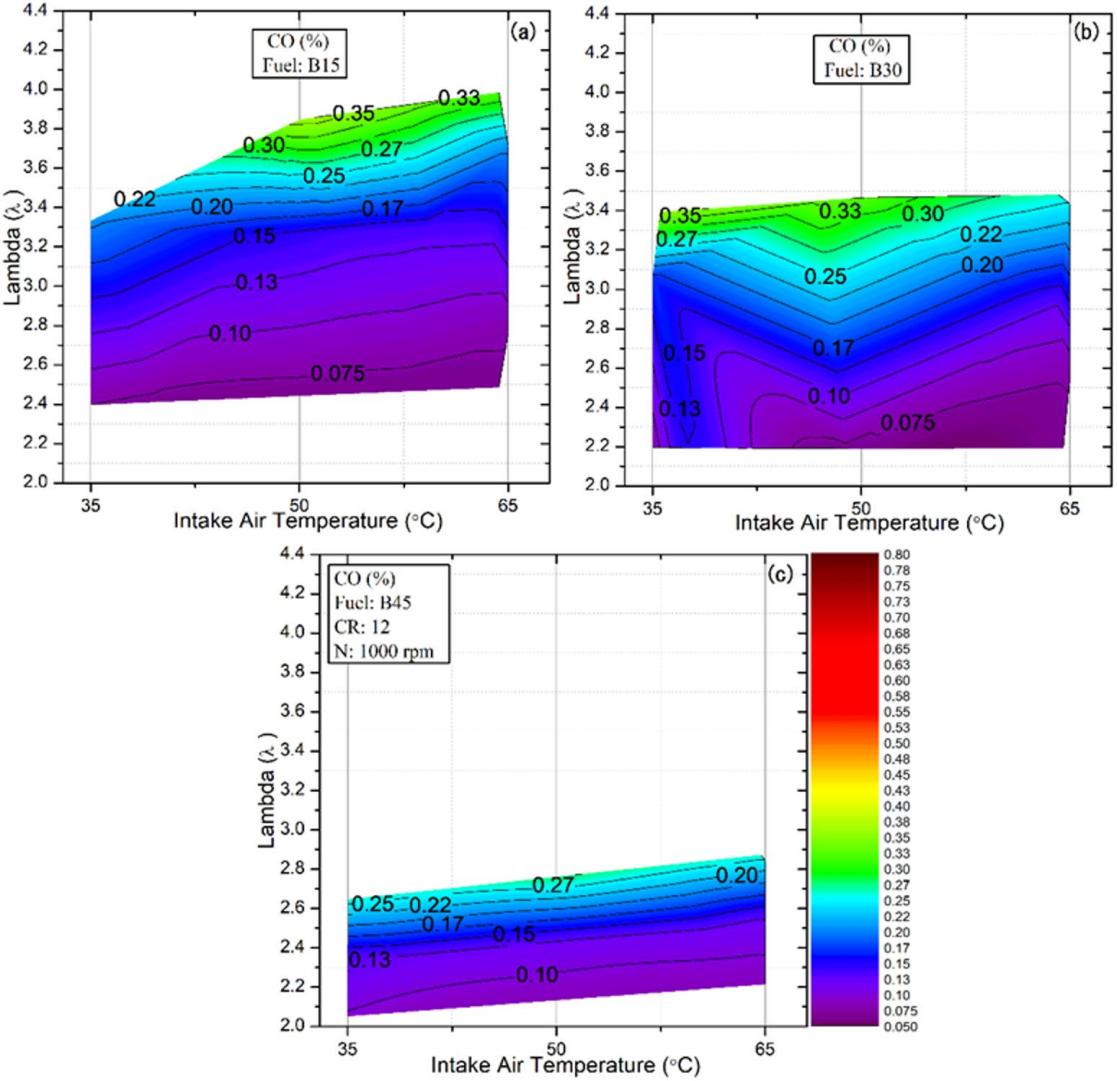
Effect of lambda and IAT on CO emissions for different fuel blends.

The formation of CO emissions is strongly influenced by the fuel’s intrinsic physical and chemical properties, including volatility, oxygen content, and molecular structure. In HCCI combustion, the extremely rapid heat release limits the time available for complete carbon oxidation. In addition, the relatively low gas temperatures at the end of combustion inhibit the conversion of CO to CO₂, thereby increasing CO emissions^73,74^. Figure 12 presents the effect of the tested fuels on CO emissions in HCCI mode under varying IAT and λ values. The results demonstrate that λ exerts a significant influence on CO emissions, as it directly governs the air–fuel ratio and thereby affects the post‑combustion gas temperature. Elevated CO levels were particularly observed at higher λ values, with the B15 blend reaching the maximum emissions. This is attributed to its broadest operating range under lean conditions, extending up to λ ≈ 4, resulting in increased CO formation across the tested fuels. This is primarily due to lower fuel concentration in the cylinder, which reduces combustion temperatures and limits oxidation reactions, thereby increasing CO levels. While lowering the lambda values led to a reduction in CO emissions across all tested fuels and IAT. This trend aligns with observations reported by Solmaz, who also found decreased CO emissions with reduced lambda values^53^.

In comparison, the influences of fuel type and IAT are less pronounced than the effect of λ, as shown in Fig. 11. Nevertheless, the results clearly demonstrate that increasing the IAT consistently reduces CO emissions, even under high‑λ operating conditions. This reduction can be attributed to the advancement of combustion phasing and the more complete combustion achieved at higher IAT, which results in elevated in-cylinder pressure and temperature. In addition, improved mixture distribution and the mitigation of the narrow-gap effect at higher IATs further promote CO oxidation. For example, for the B45 blend at λ = 2.2, CO emissions decreased from 0.107% to 0.090% and 0.084% as the IAT increased from 35 °C to 50 °C and 65 °C, respectively. Moreover, increasing the proportion of DEE in the blends contributes to a further reducing CO emissions, consistent with the trend observed for HC, which can be attributed to DEE’s inherent oxygen content, which promotes more complete combustion of the fuel^75^. Conversely, the present results indicate that, under comparable operating conditions, increasing the butanol fraction in the fuel blend further elevates CO emissions; despite the fuel’s oxygen content, butanol’s LHV reduces in‑cylinder temperatures, suppressing complete carbon oxidation^76^. For instance, at an IAT of 50 °C and λ = 2.5, CO emissions increased from 0.067% to 0.081% and 0.117% as the butanol fraction in the blends increased from 15% to 30% and 45%, respectively.

### Emissions of CO_2_

**Fig. 13:**
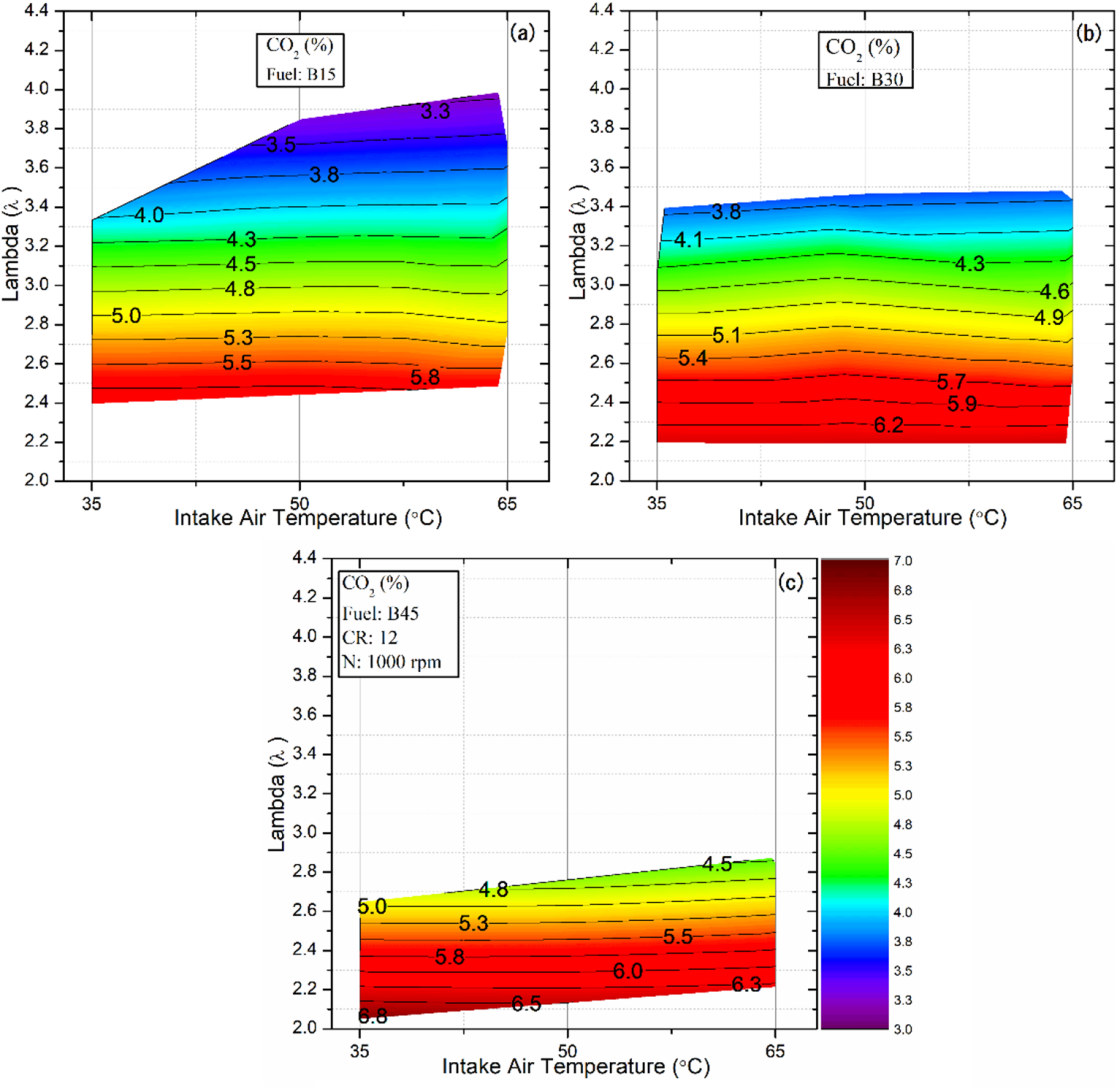
Effect of lambda and IAT on CO2 emissions for different fuel blends.

Figure 13 illustrates the variation of CO_2_ emissions under different IAT and λ conditions. The results show that a decrease in λ raises the end‑of‑combustion temperature, thereby creating favorable conditions for CO oxidation to CO₂ and, consequently, increasing CO₂ emissions. In contrast, lean mixtures provide excess oxygen, facilitating the complete combustion of the fuel carbon and promoting CO₂ formation rather than CO across all tested fuels and IAT. The highest CO₂ levels for all fuels were observed near the knock limit, whereas near the misfire limit, CO₂ emissions were lowest, and both HC and CO emissions increased. For example, under an IAT of 35 °C with the B15 blend, CO₂ emissions decreased from 5.91% to 5.46% and 5.04% as the λ value increased from 2.4 to 2.6 and 2.8, respectively. These findings are consistent with previous reports on HCCI combustion, which show that CO₂ emissions correlate strongly with lambda values^77,78^.

Furthermore, CO emissions are oxidized to CO₂ at temperatures above approximately 1500 K^79^. Consequently, a reduction in CO emissions accompanied by a slight increase in CO₂ emissions is inevitable in this study as temperatures rise. These findings contrast with the previously observed trends for CO, while consistently demonstrating that the influence of λ is more pronounced than that of temperature, as illustrated in Fig. 13. For instance, with the B15 fuel blend at a λ value of 3.33, CO₂ emissions increased from 4.02% to 4.11% and 4.25% as the intake‑air temperature rose from 35 °C to 50 °C and 65 °C, respectively. Concerning the effect of the fuel blend, no significant influence on CO₂ emissions was observed compared to the pronounced impact of engine load (λ values). Due to the narrow operating range of fuel B45 and its tendency to function predominantly at higher loads, attributable to the specific properties of butanol, an increase in CO₂ emissions was consequently recorded. Thus, the observed increase in CO₂ emissions at higher loads can be interpreted as evidence of more complete combustion, reflecting improved combustion efficiency.

## Conclusions

This study investigated the combustion, performance, and emissions characteristics of a single-cylinder HCCI engine operating on butanol/DEE blends (B15, B30, B45) under varying IAT and excess air ratios (λ). The main findings are summarized as follows:

1. Combustion Phasing and Duration: Increasing DEE content with elevated IAT and richer mixtures advanced combustion, in-cylinder pressure, and heat-release rates, whereas higher butanol fractions delayed ignition, particularly for B45, shifting CA10 and CA50 toward the expansion stroke. Combustion duration was prolonged under leaner mixtures and elevated IAT, while higher butanol fractions shortened CD.
2. Performance Metrics (IMEP and ITE): DEE addition and elevated IAT expanded the operating range by enhancing reactivity, whereas higher butanol fractions increased peak IMEP but narrowed the operational range. Increasing butanol to 45% delayed CA50 to the optimal phasing, yielding the highest indicated thermal efficiency (41.43% at 35 °C).
3. Pressure Rise and Engine Stability: Higher DEE in B15 accelerated combustion, increasing PRRmax and approaching knock limits, while higher butanol fractions effectively suppressed PRRmax and enhanced stability. Combustion stability, as measured by COV_IMEP_, improved with higher λ and butanol content, although elevated IAT slightly increased cyclic variability.
4. Emissions Behavior: HC and CO emissions generally decreased with higher IAT and DEE content but increased with elevated λ or higher butanol fraction due to lower in-cylinder temperatures and localized fuel-rich zones. CO₂ emissions followed the inverse trend, reflecting more complete combustion at higher loads and optimized phasing.

Overall, these results indicate that a balanced fuel blend with adequate DEE and controlled butanol fraction can optimize HCCI combustion, enhance performance, and minimize emissions, while highlighting the trade-offs associated with ultra-lean operation and high IAT. These findings agree with earlier studies that highlight the high reactivity of DEE^25^and the combustion-moderating effect of butanol^13^ in HCCI operation.

### Future perspectives

Future research should expand the present analysis to a wider range of engine speeds and loads and to multi-cylinder platforms to assess scalability and real-world feasibility. Coupling butanol/DEE reactivity management with advanced combustion control strategies, such as exhaust gas recirculation (EGR), variable valve timing, and boosting systems, may enable greater operational flexibility and improved stability at high load.

## Data Availability

All data generated or analysed during this study are included in this article.
